# Half a pack of cigarettes a day more than doubles DNA breaks in circulating leukocytes

**DOI:** 10.1186/1617-9625-8-14

**Published:** 2010-11-17

**Authors:** Maneli Mozaffarieh, Katarzyna Konieczka, Daniela Hauenstein, Andreas Schoetzau, Josef Flammer

**Affiliations:** 1 Department of Ophthalmology, University of Basel, Mittlere Strasse 91, CH-4031 Basel, Switzerland

## Abstract

**Background:**

The mechanisms by which smoking induces damage is not known for all diseases. One mechanism believed to play a role is oxidative stress. Oxidative stress leads to cellular damage including DNA damage, particularly DNA breaks. We conducted this study to test the hypothesis that smokers have increased DNA breaks in their circulating leukocytes.

**Methods:**

A comparative quantification of single-stranded DNA breaks was performed by comet assay analysis in the circulating leukocytes of ten healthy smokers (average smoking rate: half a pack a day, range: 9-12 cigarettes a day) and ten age and sex matched healthy non-smokers. DNA breaks lead to smaller pieces of DNA, which migrate out of the nucleus forming a tail during gel-electrophoresis. Damage of an individual cell was quantified by the parameters tail moment and olive moment.

**Results:**

Smoking had a clear effect on both study parameters (tail and olive moment). Smokers had more than double the amount of ss-DNA breaks in their circulating leukocytes than non-smokers [tail moment: 0·75 AU _[smokers] _compared to 0·2 AU _[non-smokers]_; olive moment: 0·85 AU _[smokers] _compared to 0·3 AU _[non-smokers]_; both p < 0·001].

**Conclusion:**

Smoking half a pack a day interferes with DNA integrity. One potential explanation for the enhanced DNA breaks in smokers is oxidative stress.

## Background

Little doubt exists that smoking is an important risk factor for various

Diseases [1]. Extrapolating from the tobacco-attributed mortality rates in 1995, and taking into account population growth, approximately 3·4 million deaths in developed countries from tobacco is anticipated in 2025 [2]. The exact mechanism by which smoking contributes to the pathogenesis of diseases, like cataracts and age-related macular degeneration, has not yet been identified in detail. One plausible cause is oxidative stress. The term oxidative stress is widely used in the literature but not very well defined. Oxidative stress occurs when the amount of ROS generated in cells exceeds the capacity of normal detoxification systems [3]. It leads to cellular damage, including DNA damage, in particular DNA breaks.

Under physiological conditions, DNA can undergo spontaneous breaks. DNA damage can occur as double-strand (ds) breaks or as single-strand (ss) breaks [4]. The number of DNA breaks depends on different factors. For example, it increases with age. Fortunately, DNA damage can be repaired by various mechanisms [5]. As oxidative stress accelerates DNA breaks, we hypothesized that smoking, by inducing systemic oxidative stress, would increase DNA breaks. To investigate this hypothesis we quantified ss-DNA breaks by comet assay in circulating leukocytes of healthy smokers and healthy non-smokers.

## Methods

### Subjects

Ten smokers and ten age and sex matched non-smokers were recruited after a notification at the University of Basel informed potential volunteers of the opportunity to participate in a scientific research project. Ethical approval was obtained from the local medical ethics committee, and written, informed consent was received from all subjects before admission into the study. The study was designed and conducted in accordance with the tenets of Declaration of Helsinki. The age of the volunteers was between 18 and 60 years. Subjects with any known systemic disease, for example, diabetes, were excluded. In addition, smokers had to have smoked, on average, half a pack of 20 cigarettes a day for at least a year. All subjects were without medications.

### Isolation of leukocytes

Blood samples (20 ml) anti-coagulated with heparin were obtained by venopuncture from the volunteers. The leukocytes were isolated using Ficoll-Histopaque gradients as previously described. The leukocyte bands were removed from the interface between plasma and the histopaque layers of each tube and collected into one 50 ml tube. The total volume was brought to 50 ml with cold Dulbecco's Modified Eagle Medium (DMEM, Gibco ™). The cell suspension was washed three times with DMEM and the total number of cells was determined. Cells were finally suspended in PBS and aliquoted into eppendorf tubes at 10^7 ^cells/tube.

### Comet assay (Single cell gel electrophoresis)

This simple, sensitive technique permits the detection of single stranded DNA damage in single cells when performed in alkaline conditions. This method has previously been described in detail in literature. The cells under study are embedded in agarose on a slide and subjected to lysis followed by electrophoresis under specific conditions. During electrophoresis, the damaged and fragmented negatively charged DNA migrates away from the nucleus towards the anode. The amount of migrated DNA is a measure of the extent of DNA damage. To detect DNA, the slides are stained with Sybr green and examined by fluorescence microscopy equipped with a personal computer based analysis system which enables quantification of DNA damage. Cells containing damaged DNA have the appearance of a comet with a bright head and tail (Additional file 1, Photo 1). In contrast, undamaged DNA appears as an intact nucleus with no tail (Additional file 2, Photo 2).

### Quantification of DNA breaks

It is recommended by the manufacturers of the comet microscope and imaging software (Nikon AG, Zurich, Switzerland) that 50 cells on each slide be chosen at random for the quantification of DNA damage using the computer software. Tail moment is defined as the product of the tail length and the tail DNA percentage of the total DNA [Tail moment = Tail Length × Tail DNA/100]. In addition, a function known as olive tail moment was evaluated. Olive tail moment represents the product of the distance between the centers of the mass of head and tail regions and the tail DNA percentage of the total DNA [Olive moment = (Tail mean-Head mean) × Tail DNA/100]. Tail moment and olive tail moment are calculated by the computer software system as an average for the 50 cells selected for measurement.

### Statistical Analysis

The statistical evaluation was done in two steps: first, descriptive statistics, and then a test-statistical analysis. Both were done with the parameter tail moment and olive tail moment. As both parameters were zero-inflated (had many zeros), their distribution was heavy-tailed. The assumptions for usual regression modeling were therefore violated. To overcome this problem, in the test statistics, the fractions of non-zero values compared to the total number of observations were counted for each subject. These fractions were approximately normally distributed. T-tests were performed to compare the results of smokers to non-smokers. A p-value < 0·05 was considered significant. All evaluations were performed using the SPSS statistical package, R version 2·8·1.

## Results

**Figure 1:**
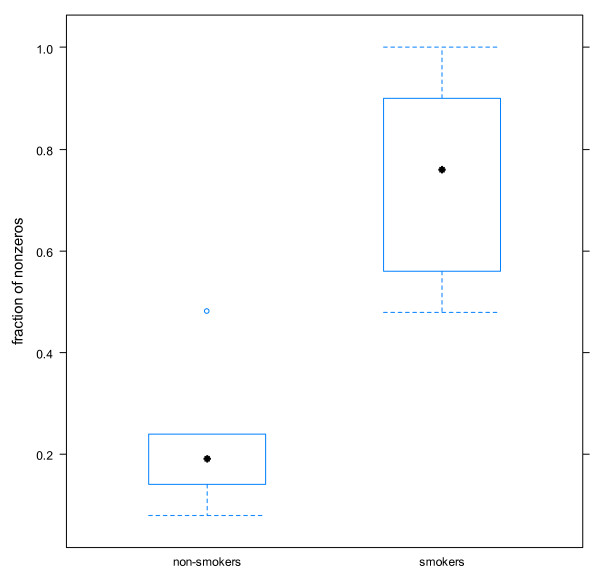
**Tail moment assessed by comet assay**. The fraction of non-zeros as measured by the parameter tail moment assessed by comet assay. Smokers had more than double the fraction of non-zeros compared to non-smokers.

Smoking had a clear effect on both study parameters (tail and olive moment) (Figure 1). Smokers had a significantly higher amount of ss-DNA breaks in their circulating leukocytes than non-smokers [tail moment: 0·75 AU _[smokers] _compared to 0·2 AU _[non-smokers]_; olive moment: 0·85 AU _[smokers] _compared to 0·3 AU _[non-smokers]_; both p < 0·001]. Table 1 presents the results of the descriptive statistics as well as the differences of means between the study groups, with corresponding 95% confidence intervals and p-values.

**Table 1:** Results of the descriptive statistics and test statistics

Descriptive statistics	Tail moment			Olive moment		
**Group**	**non-smokers**	**smokers**	**ALL**	**non-smokers**	**smokers**	**ALL**
**Mean**	0·40	9·37	4·82	0·24	5·06	2·61
**Median**	0·00	0·44	0·00	0·00	0·54	0·03
**StdDev**	3·43	18·26	13·79	1.74	10.19	7.65
**IQR**	0·00	9·46	0·83	0·03	4·54	0·76
**Min**	0·00	0·00	0·00	0·00	0·00	0·00
**Max**	52·97	115·24	115·24	27·22	62·88	62·88
**N**	500	485	985	500	485	985
**Test statistics**	**Tail moment**			**Olive moment**		

Differences of Means*	0.54			0.48		
**95% C.I**.	0·39-0·68			0·32-0·64		
**P-value**	< 0.001			< 0.001		

The descriptive statistics (based on calculation of raw data) of the tail moment and olive moment in smokers and non-smokers is shown in the upper section of the table. The results of the test statistical analysis in which fractions of non-zero values were compared to the total number of observations have been shown in the lower part of the table.

IQR: Inter-quartile range

* Fraction of nonzeros

## Discussion

Our findings suggest that smokers have a significantly higher rate of ss-DNA breaks than non-smokers. ss-DNA breaks can result from a variety of factors including UV light [6], X-rays [7], ionizing radiation[8], toxins [9], chemicals[10], or by reactive oxygen species (oxidative stress) resulting as by-products of normal metabolic processes [11]. DNA breaks are increased in different human cell cultures when exposed to cigarette smoke [12-15]. Increased DNA breaks have previously also been detected in the leukocytes of smokers [16]. This increase in DNA breaks of leukocytes is not related to the amount of cigarette tar inhaled [17,18].

The increased number of breaks could either be due to an increased incidence of breaks or a decreased repair capacity or both. Theoretically, oxidative stress may give an explanation for both an increased incidence of breaks as well as a decreased repair capacity.

There are several indications pointing towards an increased incidence of oxidative stress in smokers. Oxidative stress in our cells is caused by an imbalance between the production of reactive oxygen species (ROS) and our biological system's ability to neutralize ROS and repair the resulting damage such as DNA breaks [19]. Cigarette smoke contains molecules that act as potent carcinogens (eg. benzo[a]pyrene)[20], as well as a large amount of ROS forming substances such as catechol or hydroquinone [21]. These substances enhance free radical mediated reactions. The actual mechanism's by which smoking induces damage is not entirely known. Various studies support the view of an increased oxidative stress in smokers. Examples include oxidative modifications on muscle proteins [22], oxidative DNA damage in lung tissues [23] as well as in human tracheal smooth muscle cells [24]. Under normal conditions, the ss-DNA breaks are repaired approximately within an hour[25]. It is possible that certain polymorphisms, by affecting DNA repair capacity, enhance the risk for smoking related diseases[26-28].

Smoking also plays an important role in eye diseases. Smokers have, on the average, a higher intraocular pressure [29], cataract at earlier ages [30] and a higher risk for arterial/venous occlusions [31] as well as for age-related macular degeneration (AMD) [1]. Smokers particularly suffer from the more severe form of AMD, namely exudative (wet) AMD [32].

In summary, smoking half a pack a day more than doubles ss-DNA breaks. We assume the increased number of DNA breaks in leukocytes of smokers to be due to an increased oxidative stress. Further investigations on the role of oxidative stress and DNA repair capacity may have implications for understanding the mechanisms by which smoking induces damage.

## Competing interests

The authors declare that they have no competing interests.

## Authors' contributions

MM recruited volunteers, participated in comet assay analysis and drafted the manuscript. KK carried out comet assay analysis in the laboratory. DH quantified DNA damage. AS performed the statistical analysis. JF conceived of the study, and participated in its design and coordination and helped to draft the manuscript.

## Supplementary Material

Additional file 1 **Photo 1**. The cells of the smokers analyzed by comet assay analysis. Each spot represents the DNA of an individual cell. The less bright green "comet-shaped" area adjacent to the nucleus (arrow) represents DNA breaks that are small enough to move in the gel. Click here for file

Additional file 2 **Photo 2**. The cells of the non-smokers analyzed by comet assay analysis. Each spot represents the DNA of an individual cell. The bright green, round spots represent intact DNA. Intact DNA is a large molecule that does not migrate much in the electrophoretic field. Click here for file
