# Supplementary figures and images for: Half a pack of cigarettes a day more than doubles DNA breaks in circulating leukocytes
Source: Tob Induc Dis. 2010 Nov 17;8(1):14. doi: 10.1186/1617-9625-8-14 (PMC2996352; doi:10.1186/1617-9625-8-14)

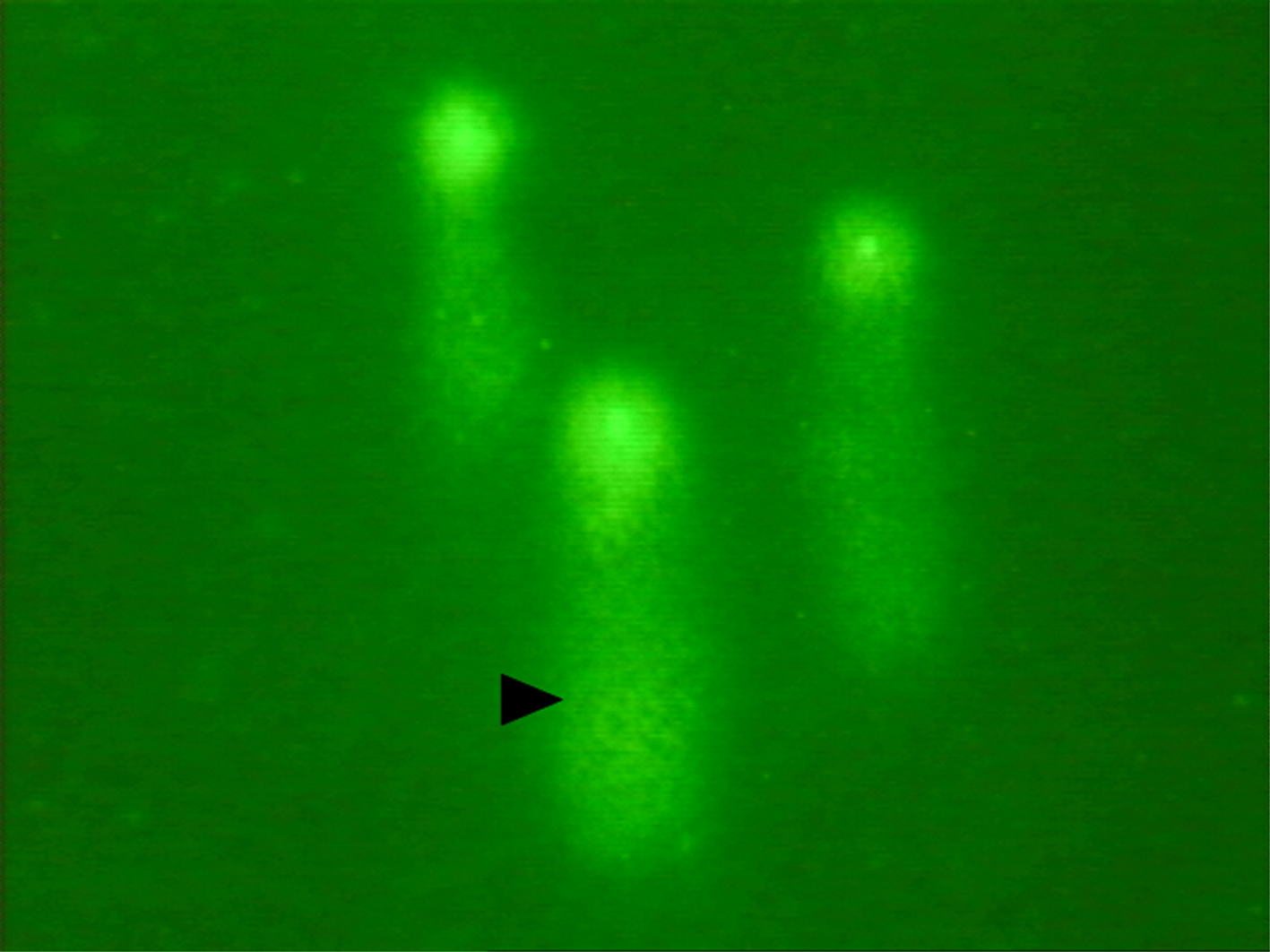

Supplement: Additional file 1 — Photo 1. The cells of the smokers analyzed by comet assay analysis. Each spot represents the DNA of an individual cell. The less bright green "comet-shaped" area adjacent to the nucleus (arrow) represents DNA breaks that are small enough to move in the gel. [file 1617-9625-8-14-S1.TIFF]

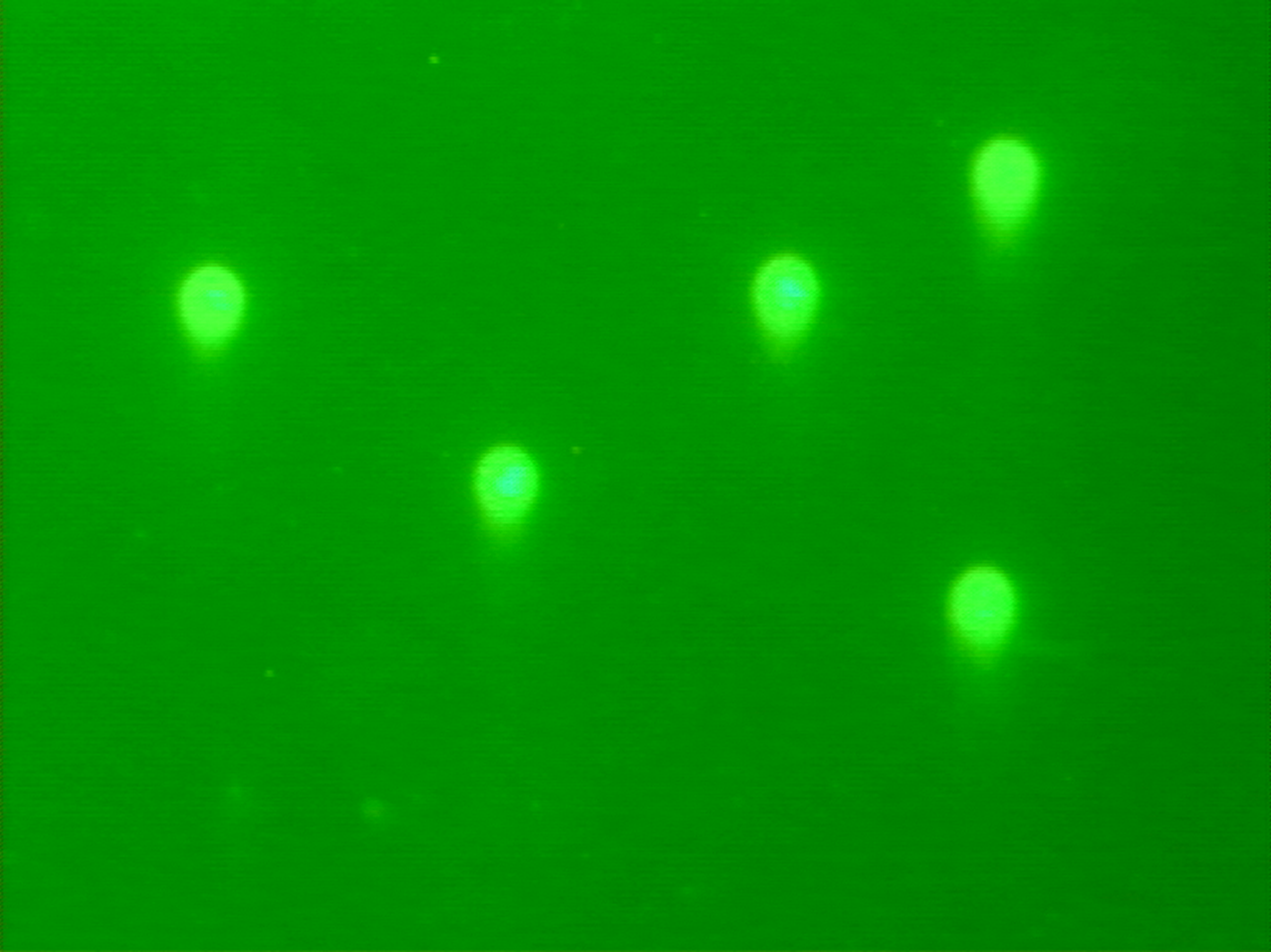

Supplement: Additional file 2 — Photo 2. The cells of the non-smokers analyzed by comet assay analysis. Each spot represents the DNA of an individual cell. The bright green, round spots represent intact DNA. Intact DNA is a large molecule that does not migrate much in the electrophoretic field. [file 1617-9625-8-14-S2.TIFF]
